# Editorial: Ethical and psychiatric considerations in euthanasia and medically assisted suicide (E/PAS)

**DOI:** 10.3389/fpsyt.2026.1910723

**Published:** 2026-06-29

**Authors:** Christian G. Huber, Johannes M. Hennings, Manuel Trachsel

**Affiliations:** 1 Universitäre Psychiatrische Kliniken (UPK) Basel, Klinik für Erwachsene, University of Basel, Basel, Switzerland; 2 Department of Psychiatry and Psychotherapy, Ludwig-Maximilians-University Hospital, Munich, Germany; 3 Department of Dialectical Behavioral Therapy, Kbo Clinic Region Munich, Haar, Germany; 4 Clinical Ethics Unit, University Hospital Basel (USB), and Universitäre Psychiatrische Kliniken (UPK) Basel, Basel, Switzerland; 5 Faculty of Medicine, University of Basel, Basel, Switzerland

**Keywords:** assisted dying, assisted suicide, decision making capacity, euthanasia, medial ethics, mental illness, psychiatry, stigmatization

In mental health care, suicidality, i.e., not being willing to continue with one’s own life, is one of the most altering symptoms occurring in various psychiatric conditions. In these cases, the role of healthcare professionals is to protect the person from the risk of suicide and ensure adequate treatment to address the underlying illness (1–3). However, just like in non-psychiatric disorders, there are persons who have received and exhausted state-of-the-art, evidence- and guideline-based treatment and still don’t reach sufficient recovery and quality of life, causing subjectively intolerable suffering without a plausible perspective for improvement (3). Furthermore, the distinction between non-psychiatric and psychiatric illnesses in this context may not be completely clear, as mental health issues are often an important comorbidity in somatic illnesses leading to a wish for assisted dying (4). And, in both cases, suicidal symptoms (such as suicide ideations or suicide attempts) can be considered psychologically as a means to mentally reduce the individual suffering (e.g., showing a possible way out if other coping strategies are not available) (5).

The question of whether and under which circumstances a medical ill person should be able to receive euthanasia (i.e., by an active deadly intervention) or physician-assisted suicide (i.e., medically provided means to kill oneself) is a significant and contested matter from a bioethical as well as medical perspective debated worldwide. In the case of a mental illness, the situation may be considered even more complex since the wish to die can occur as an inherent part of the psychiatric disease’s pathology (6).

Western countries exhibit diverse psychiatric regulations and practices concerning euthanasia and medically assisted suicide (E/PAS). While some nations permit these practices under specific conditions associated with unbearable psychiatric suffering or treatment-resistant mental health disorders, others haven’t extended them to these disorders yet, allowing E/PAS only under certain medical conditions (4, 7). Evaluating the mental decision-making capacity of patients who choose E/PAS and defining ‘unbearable psychological distress’ are central psychiatric issues in this practice (8). Additionally, the ethical implications of extending E/PAS to minors and individuals with neurological disorders or those depending on life-support treatments uncovers another area warranting comprehensive scrutiny (7). Furthermore, people with mental disorders are often stigmatized (9–12). It therefore seems plausible that acceptance of assisted dying by healthcare professionals, within the legal system and in the general population, might also be related to stigmatization (13).

Building on a previous special issue on assisted dying in persons with mental illness from 2022 (14), this Research Topic seeks to delve into the evolving psychiatric approaches and policies regarding E/PAS in the recent literature.

Six of the 14 papers published within the scope of the current Research Topic focus on country-specific legal and regulatory perspectives. Bosma et al. analyzed publicly available data from the regional euthanasia review committees in the Netherlands, where both euthanasia and assisted suicide for persons with mental health illnesses are legally possible, to gain insight into the country-specific practices. Following up, Verhofstadt et al. examine the practices for euthanasia and physician-assisted dying in the Netherlands and Belgium, focusing in particular on younger persons requesting E/PAS because of a mental health condition and on the provision of adequate psychiatric care. Gloeckler et al. compare the Netherlands’ regulations on E/PAS for mental health illnesses with those in Switzerland and Germany, including also the perspective of advance directives.

Gupta and Downie provide their conceptual analysis on the current situation in Canada, where access to medically assisted dying is possible for persons with an incurable illness. The authors explore what this means for persons with a mental illness.

Lastly, two papers focus on the situation in Italy. Turillazzi et al. provide a policy and practice review on the current regulations, discuss the absence of specific regulations on E/PAS, describe the current public debate and present possible future directions. Albore et al. focus on the country-specific regulation constraining assisted suicide to dependence on life-sustaining treatments.

Three more publications look at the ethical perspectives of E/PAS for mental health conditions. Goldblatt et al. examine how to balance between suicide prevention and providing assisted suicide to those facing intolerable suffering. They also look at the potential alleviation of suffering for the person requesting assisted dying while addressing the distress for family members and healthcare professionals because of E/PAS for a person they took care for. Tripi et al. review the international literature on clinical, ethical, and medico-legal aspects of E/PAS. In their perspective paper, they describe which domains are important for E/PAS assessment, discuss the persisting challenges, and recommend structured and multidisciplinary evaluation processes to promote the discussion. Finally, van Vlerken et al. examine the complex question if and under which circumstances, after physician-assisted suicide in persons with a mental illness, organ donation should be allowed and performed.

Three further publications provide insight into clinician and nurse perspectives. Bachmetjev et al. analyze the views of physicians from Lithuania on E/PAS and further end-of-life decisions, also focusing on persons with mental illnesses, and explore how they are influenced by demographic variables, religious beliefs, and professional experience. Jonsson et al. report on twelve semi-structured interviews with physicians from Sweden, exploring their views and experiences with issuing certificates for assisted dying. In addition, Seidlein et al. provide a view on the ethical perspective for nurses supporting people in long-term care.

Adding knowledge in the situation of E/PAS in specific diagnosis groups, Roff and Cook-Cottone discuss physician-assisted dying for people with eating disorders. They provide a systematic review of the international literature, supplemented by government reports, and discuss reporting gaps regarding mental health issues.

Finally, Pürcher et al. look at media coverage of PAS. The authors analyze newspaper coverage of suicide in the time periods before, during and after legalization of assisted suicide in Austria in 2022.

**Table 1** gives an overview of the papers published within the scope of the current Research Topic sorted by main topic.

**Table 1:** Overview on the papers published within the scope of the research topic.

Topic/authors	Title	Article type
Country-specific legal and regulatory perspectives		
Albore et al.	Reconsidering dependence on life-sustaining treatment as a criterion for assisted suicide: the Italian legal unicum in comparative perspective	Perspective
Bosma et al.	The Dutch practice of euthanasia and assisted suicide in patients suffering from psychiatric disorders: a qualitative case review study	Original Research
Gloeckler et al.	Differential treatment of individuals with mental health conditions in high-consequence decision-making: a comparison of policy on advance directives and assisted suicide in three European countries	Policy and Practice Reviews
Gupta and Downie	Interpreting and operationalizing the incurability requirement in Canada’s assisted dying legislation	Conceptual Analysis
Turillazzi et al.	Physician-assisted suicide in Italy: where do we stand and where do we want to go?	Policy and Practice Reviews
Verhofstadt et al.	Exploring the interplay of clinical, ethical and societal dynamics: two decades of Medical Assistance in Dying (MAID) on psychiatric grounds in the Netherlands and Belgium	Hypothesis and Theory
Ethical perspectives		
Goldblatt et al.	Exploring multiple levels of suffering and suicide prevention in an era of emerging national legislations	Perspective
Tripi et al.	Forensic-Psychiatric and Medico-Legal Challenges in Euthanasia and Assisted Suicide for Patients with Mental Disorders: Balancing Autonomy, Vulnerability, and Professional Responsibility	Perspective
van Vlerken et al.	Organ donation after medically assisted death on psychiatric grounds: an ethical analysis	Perspective
Clinician and nurse perspectives		
Bachmetjev et al.	Demographic influences on Lithuanian physicians’ attitudes toward medical assistance in dying: a cross-sectional study	Original Research
Jonsson et al.	Views and experiences on writing certificates for assisted dying: interviews with Swedish physicians	Original Research
Seidlein et al.	Nursing ethical dimensions of euthanasia and medically assisted suicide for older people in need of long-term care	Perspective
E/PAS in specific diagnosis groups		
Roff and Cottone	Assisted death in eating disorders: a systematic review of cases and clinical rationales	Systematic Review
Media coverage		
Pürcher et al.	Media portrayals of assisted suicide before, during, and after legalization changes: content analysis of the reporting in Austrian newspapers	Original Research

Most of the submissions were theoretical papers, with one systematic review, two policy and practice reviews, a conceptual analysis, a hypothesis and theory paper, and five perspective papers. This might be an indication that the field is focused on the discourse on actual policies and their future development, and that data-based, empirical research is still underdeveloped.

In the Research Topic description, the editors invited submissions evaluating the prevalence of patients with mental disorders, with particular focus on persons diagnosed with severe and persistent mental illness (SPMI, e.g., schizophrenia spectrum disorder, unipolar depression or bipolar disorder, severe personality disorders), and with neurological diagnoses (e.g., dementia, autism spectrum disorder). However, only one paper focused on a specific diagnosis (eating disorders). Further absent topics were studies providing insight into cognitive mechanisms that may influence patients’ decisions to access E/PAS, regardless of the presence of mental and/or neurological disorders. Also, there were no papers with a focus on alternative strategies to offer to individuals requesting access to E/PAS for mental disorders sketching out the latest developments in psychiatric and/or neurological fields in this regard. In addition, there were no submissions on refining the standardization of procedures used in the psychiatric evaluation of patients seeking for E/PAS, including research on refining and validating procedures and assessment tools for evaluating mental capacity to make decisions about assisted dying, defining and assessing ‘unbearable and unrelievable mental distress’, and establishing criteria for ‘absence of prospects for improvement’. This list could therefore serve as an inspiration for research that is still needed in the field.

Nevertheless, the considerable number of papers collected shows that this is a broad and active research field in psychiatry, and that there is rising awareness in academic and clinical psychiatry. Besides legal considerations, the discussion about E/PAS also reveals significant yet unsolved dualities that challenge health care providers during their everyday work, especially in the field of mental health, such as suicide prevention vs. the “right to die” or keeping the role of the undoubtful advocate of life vs. limited means to reduce individual suffering. With the presented article collection, we aim to allow various perspectives on the polarizing topic E/PAS to stimulate further fruitful discussions.
